# Single-Trial Electroencephalography Discrimination of Real, Regulated, Isometric Wrist Extension and Wrist Flexion

**DOI:** 10.3390/biomimetics10030187

**Published:** 2025-03-18

**Authors:** Abdul-Khaaliq Mohamed, Vered Aharonson

**Affiliations:** 1 School of Electrical and Information Engineering, University of Witwatersrand, Johannesburg 2050, South Africa; 2 Department of Basic and Clinical Sciences, Medical School, University of Nicosia, Nicosia 2421, Cyprus

**Keywords:** brain–computer interface, wrist extension, wrist flexion, electroencephalography (EEG)

## Abstract

Improved interpretation of electroencephalography (EEG) associated with the neural control of essential hand movements, including wrist extension (WE) and wrist flexion (WF), could improve the performance of brain–computer interfaces (BCIs). These BCIs could control a prosthetic or orthotic hand to enable motor-impaired individuals to regain the performance of activities of daily living. This study investigated the interpretation of neural signal patterns associated with kinematic differences between real, regulated, isometric WE and WF movements from recorded EEG data. We used 128-channel EEG data recorded from 14 participants performing repetitions of the wrist movements, where the force, speed, and range of motion were regulated. The data were filtered into four frequency bands: delta and theta, mu and beta, low gamma, and high gamma. Within each frequency band, independent component analysis was used to isolate signals originating from seven cortical regions of interest. Features were extracted from these signals using a time–frequency algorithm and classified using Mahalanobis distance clustering. We successfully classified bilateral and unilateral WE and WF movements, with respective accuracies of 90.68% and 69.80%. The results also demonstrated that all frequency bands and regions of interest contained motor-related discriminatory information. Bilateral discrimination relied more on the mu and beta bands, while unilateral discrimination favoured the gamma bands. These results suggest that EEG-based BCIs could benefit from the extraction of features from multiple frequencies and cortical regions.

## 1. Introduction

Wrist extension (WE) and wrist flexion (WF) movements are crucial to stabilise, position, and control the hand [1,2]. This, in turn, enables people to perform activities of daily living, such as writing, opening a jar, donning pants, perineal cleansing, and drinking from a cup [3,4]. Patients undergoing post-stroke rehabilitation perform key exercises involving WE and WF to regain full hand and wrist motion [5]. A robotic prosthetic hand capable of performing WE and WF could enable upper limb amputees to perform activities of daily living more naturally [6,7,8].

A brain–computer interface (BCI) capable of controlling a bionic hand (robotic prosthetic or orthotic hand) can assist people with motor impairments in regaining basic hand functionality [9,10,11]. The controlled bionic hand should be capable of performing WE and WF, while the controlling BCI should be able to interpret the neural control signals for these movements. For BCI applications, electroencephalography (EEG) is the most widely used bio-signal from which neural hand control signals are extracted [12].

EEG-based BCI studies have aimed to improve the interpretation of neural signals associated with WE and WF [13]. Interpretation is enabled by classifying or discriminating between relevant EEG signal features associated with two or more classes of motor tasks. From the multitude of possible discriminations between motor task classes, we considered two types of discrimination investigations involving WE and WF motor tasks. These are (1) discrimination between right-hand (RH) and left-hand (LH) motor tasks and (2) discrimination between unilateral hand motor tasks. Discrimination between right- vs. left-hand motor tasks has been investigated extensively in the BCI literature [14]. Discrimination between unilateral hand motor tasks is necessary to progress towards natural and intuitive control of a dexterous bionic hand [15]. Unilateral discrimination is more challenging, since these motor tasks activate similar areas of the motor cortices. Thus, the signal features are not spatially separable—as is the case with motor tasks from separate limbs [16,17]. Table 1 summarises studies similar to the two types of investigations mentioned above. These are denoted as right vs. left investigation (RLI) and extension vs. flexion investigation (EFI), respectively.

The studies in Table 1 involved imagined and/or real WE and WF motor tasks. BCI studies aimed at interpreting motor imagery may, in the future, enable users without hand movement capability to control a bionic hand [11,15]. The interpretation of real hand movements may be applied to BCI users with residual hand movement control, particularly those undergoing neuromuscular rehabilitation [15]. Furthermore, analysis of real movements allows parameters such as force, speed, and range of motion to be regulated, thus isolating their associated neural signal patterns from those related to kinematic movement differences [24]. This could improve the EEG interpretation of hand movement control.

Two studies from Table 1 classified signal features associated with RH and LH groupings of WE and WF imagery [13,20], but no similar study was found involving real versions of these movements. Three studies from Table 1 investigated EEG feature discrimination associated with unilateral WE and WF motor imagery [17,20,22]. Only one of these studies also included real WE and WF movements [17]. In this study, however, the real movements were not regulated with respect to force, speed, and range of movement. Hence, the two binary classification problems (RLI and EFI)—involving real, regulated, isometric WE and WF movements—have not been explored to date.

**Figure 1:**
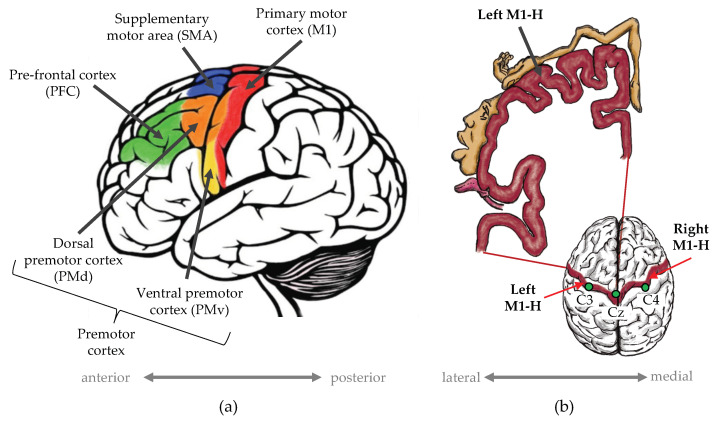
(**a**) Some regions of interest (ROIs) and (**b**) homunculi of M1 [27,41,42,43,44]. C3, C4, and Cz denote electrode positions according to the 10–20 system [45].

In the BCI studies that aimed to classify different types of hand movements, features were frequently extracted from sensorimotor rhythms within the mu and beta frequency bands [14,15]. These oscillations are believed to originate from the hand homunculus of the primary cortex (M1-H shown in Figure 1) [10,14,25]. However, a growing body of research indicates that hand movements are controlled by a combination of distributed brain regions [26,27,28,29,30,31]. In addition to M1-H, these regions of interest (ROIs) include the ventral premotor cortex (PMv), the supplementary motor area (SMA), and the prefrontal cortex (PFC), which are shown in Figure 1. Studies have also provided evidence that the ROIs communicate using a range of synchronous oscillations to achieve hand motor control [32,33,34,35]. These oscillations are not confined to the mu and beta rhythms [31]. For most (six out of nine) of the wrist movement studies shown in Table 1, the mu and beta bands produced prominent features. However, Table 1 also indicates that the delta, theta, low-, and high-gamma bands have facilitated the classification and interpretation of real and imagined WE and WF motor tasks. These bands have shown significance in the control of other hand or arm movements [36,37,38,39,40]. It is thus likely that signal features extracted from only the mu and beta frequency bands, or from M1-H only, are likely to encapsulate only a limited portion of the WE and WF motor control information. Utilising features from the full range of EEG frequencies and from multiple ROIs could encapsulate hand and wrist neuromotor control more completely. This could lead to improved EEG-based BCI performance for bionic hand control.

The primary objective of our study was to test the discrimination of real, regulated, isometric WE and WF motor tasks using two single-trial EEG-based BCI investigations: the RLI and EFI. Our secondary objective was to validate that neuromotor control patterns—which differentiate the kinematic differences between these motor tasks—include information from the delta, theta, mu, beta, low-gamma, and high-gamma frequencies, as well as from M1-H, PMv, SMA, and PFC.

## 2. Materials and Methods

**Figure 2:**
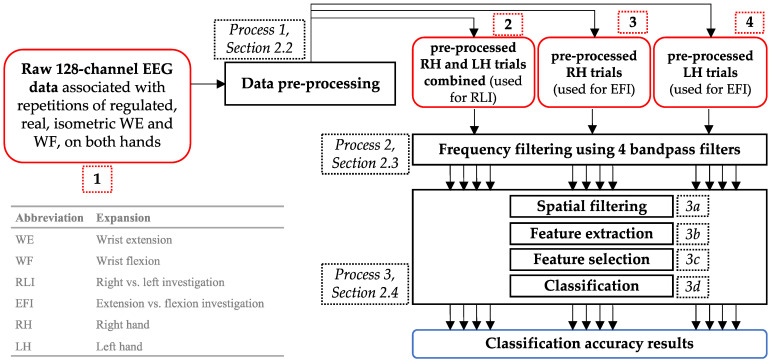
Overview of the methodology. Coloured blocks represent different aspects of the methodology: red indicates datasets, black signifies processes, and blue denotes the results. This approach was individually applied to each participant. Each process block references the section number corresponding to a comprehensive description of the process. The abbreviations used in the block text and their expansions are provided in the legend located in the bottom right corner.

The methodology of our study is outlined in Figure 2 and included signal-processing and classification methods from our previous studies [21,46,47,48]. The methods were applied to EEG data recorded with our low-cost dynamometer—the IsoReg [24]. The analysis was performed per participant, which was necessary due to the large inter-participant variances that typically occur in EEG data [10]. This yielded 12 discrimination accuracy values (4 frequency bands × 3 datasets) for each participant. MATLAB 2019b (The Mathworks Inc., Natick, MA, USA) and EEGLAB (a MATLAB plugin) [49] were used to implement the methods.

### 2.1. EEG Data Acquisition

The analysis was performed on high-resolution, 128-channel EEG data, shown as red block 1 in Figure 2. The experimental data-recording protocol, the participants, and the equipment were described in our former report [24]. Table 2, as well as the remainder of this section, provides an overview of the data acquisition procedure. This contextualises the subsequent explanation of the analysis and classification methods presented in this article.

**Table 2:** Summary of the equipment and protocols for EEG data acquisition.

EEG system manufacturer	Brain Products actiCHamp amplifier (Brain Products GmbH, Gilching, Germany).
Description of EEG channels	128 active electrodes arranged according to the 10–5 system.
Description of participants	14 volunteers (8 males and 6 females)—all right-handed, healthy, with no prior wrist injuries and BCI training, between the ages of 20 and 30 years.
Brief motor task description	Real, isometric WE and WF, sustained at 15% of their respective maximum voluntary contractions (MVCs). Each movement repetition involved four stages or time segments (S1–S4) separated by four visual instructions (“Get Ready”, “Start Moving”, “Hold Movement”, and “Stop”). The timing and visual cues are shown in Figure 3.
Number of movement repetitions for each participant	200 WE and 200 WF movements for RH. Same for the left hand. Each repetition formed a single trial.
EEG data sampling rate	500 Hz.

**Figure 3:**
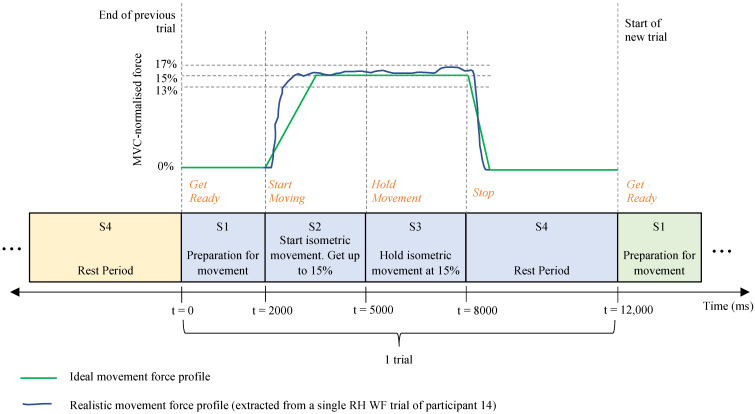
Timing diagram of a single trial common to all participants (adapted from [24]). Visual cues are shown in orange text. During S2, the participants tried to increase the relative force of either WE or WF up to 15% as fast as possible. During S3, the participants tried to sustain the isometric movement at 15%, but maintaining a relative force between 13% and 17% was deemed acceptable.

The data acquisition procedure utilised a purpose-designed force dynamometer—the IsoReg—to regulate the repetitions of WE and WF movements by controlling force, speed, and range of motion [24]. EEG was recorded while the participants were seated in a comfortable chair, facing a computer screen, with their right or left forearm fixed into the IsoReg. The IsoReg restricted hand movements to isometric WE and WF in a standardised experimental procedure, thus controlling their speed and range of motion. It measured movement force using a dual-load cell system that calculated the percentage of maximum voluntary contraction, which it displayed to help users control movement force. The measured forces were recorded and analysed relative to each participant’s respective maximum voluntary contractions (MVCs). In our former report, we verified the linearity and accuracy of force measurements and the degree of force regulation [24]. The data for LH movement repetitions for participants 13 and 14 were not usable due to technical difficulties and time constraints experienced during the recording process.

### 2.2. EEG Pre-Processing

The pre-processing of the raw EEG channel data is shown in process 2 in Figure 2. The data were inspected for excessive noise contamination by identifying channel amplitudes greater than 100 µV—the typical maximum amplitude of scalp EEG [50]. No channels were found to have high levels of noise contamination, probably due to the active filters on the electrodes of the Brain Products EEG system.

EEG channels were assigned 3D spherical coordinates corresponding to the 10–5 electrode placement system (Easy-cap, Herrsching, Germany). These coordinates enabled effective spatial filtering, which is described in Section 2.4.

The continuous data signals were divided into single trials, each with a duration of 12,000 ms. The timing paradigm of a single trial is shown in Figure 3. Trials were segmented using the rest period (S4) to avoid segmentation during movement and movement preparation (S1–S3). The “Get Ready” event in the data acquisition [24] designated t = 0 ms. Each trial started 3000 ms before the “Get Ready” event and ended 1000 ms after the “Stop” event/command [23]. The trials were corrected using the interval from t = −3000 ms to t = 0 ms as a baseline to align the rest state (where no movement occurred during S4) with the zero-volt level [17,51,52]. Data not contained within these designated trial batches were excluded from the analysis.

Digital filters were then applied to the single-trial data to remove noise while retaining most of the EEG frequencies. The filters included the following:

1. A high-pass filter at 0.5 Hz to remove DC shifts while aiming to retain most of the delta band frequencies (0–3.5 Hz);
2. A low-pass filter at 99 Hz to remove high-frequency noise above 100 Hz without removing high-gamma information (51–90 Hz);
3. A notch filter between 49 and 51 Hz to remove AC line noise.

The filtered, single-trial data underwent independent component analysis (ICA) using EEGLAB’s implementation of the extended Infomax algorithm—Runica [49]. This algorithm aimed to isolate independent components (ICs) representing contaminating artifacts from ICs representing cortical sources of neural signals. All ICs were visually inspected to search for electrooculogram (EOG) artifacts from eye blinks and eye movements; and electromyographic (EMG) artifacts from tongue, face, neck, and shoulder movements [53]. ICs that represented EOG and EMG artifacts were manually identified and removed. Thereafter, the time-series, single-trial data were reconstructed from the reduced set of ICs. The presence of motor control information in the remaining ICs was explored using the methods described in Section 2.4. This followed the bandpass filtering stage, which is described in Section 2.3.

Artifact removal was carried out according to the guidelines provided by Chaumon et al. (2015) [54]. EOG artifact ICs present themselves in the frontal part of the head (sometimes bilaterally near the eyes). They have comparatively large signal amplitudes, have peak powers in lower-frequency bands, and can exhibit peaks occurring at time intervals correlating to eye blinks. EMG artifact ICs usually have a narrow spatial focus on the sides of the head, have relatively high power in higher frequencies, and appear noisy for the duration of the trials.

Three sets of data were created from the artifact-free data. The first set contained 400 LH and RH trials and was used for the RLI. The second and third sets were used for the EFI and consisted of 200 RH trials and 200 LH trials, respectively. The three sets are illustrated in the red blocks 2, 3, and 4 of Figure 2.

### 2.3. Bandpass Frequency Filtering

As shown in Table 1 (and explained in Section 1), previous similar studies have extracted features from varying bands spanning the full EEG spectrum. Hence, in this study, we explored features extracted from the full EEG frequency spectrum, i.e., 1–90 Hz. We split the spectrum into four passbands by applying four bandpass filters to the pre-processed, artifact-free, single-trial data, as shown by process 2 in Figure 2. Based on the typical EEG frequency band descriptions [19,55,56,57,58], the bandpass filters were defined as follows: 1–7 Hz, to include delta and theta bands; 7–35 Hz, to include mu and beta bands; 35–49 Hz, to include the low-gamma band; 51–90 Hz, to include the high-gamma band. This filtering yielded 12 single-trial, bandpass-filtered datasets for each participant (four sets for the RLI and eight sets for the EFI).

### 2.4. Spatial Filtering, Feature Extraction, Feature Selection, and Classification

**Figure 4:**
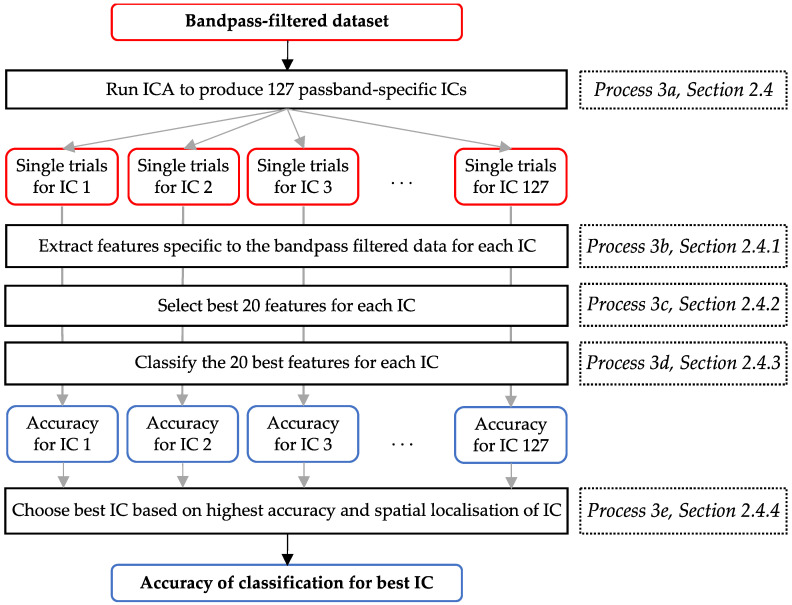
This method was applied to each of the 12 bandpass-filtered datasets of each participant. It combines the spatial filtering, feature extraction, feature selection, and classification processes. The red, black, and blue blocks, respectively, indicate datasets, processes, and results.

These processing steps pertain to process 3 in Figure 2 and are detailed in Figure 4. Our study aimed to interpret the kinematic differences between regulated WE and WF by considering a complete picture of hand motor control. Hence, signals emanating from seven ROIs were considered and approximated. These ROIs included bilateral M1-H, bilateral PFC, bilateral PMv, and the SMA, which are shown in Figure 1. We isolated frequency band-specific signals that approximated neural activity from these ROIs and evaluated the accuracy of their discrimination.

In the pre-processing stage (process 1 in Figure 2), we used ICA to remove artifacts. We used ICA again as a spatial filter to approximate and isolate signals emanating from the seven abovementioned ROIs. This is shown by process 3a in Figure 4. ICA was implemented using the Infomax ICA algorithm [49] to decompose each of the 12 datasets of bandpass-filtered, single-trial data from each participant into 127 ICs. This process generated ICs that were specific to a given participant; their RH, LH, or both hands; and a frequency range. It was, thereafter, necessary to determine which IC best represented motor control information emanating from the ROIs.

Features from each IC were extracted, selected, and classified as described in Section 2.4.1, Section 2.4.2, and Section 2.4.3, respectively, and correspond to processes 3b, 3c, and 3d in Figure 4. The resulting classification results were used in conjunction with a method of visual inspection—described in Section 2.4.4 and shown as process 3e in Figure 4—to determine the best-performing IC and its classification accuracy for each bandpass-filtered dataset. The accuracy values were then compared with the values obtained in previous WE and WF EEG BCI studies (shown in Table 1). Accuracies similar to those found in the literature suggest that the selected features encapsulated motor control information.

#### 2.4.1. Feature Extraction

**Figure 5:**
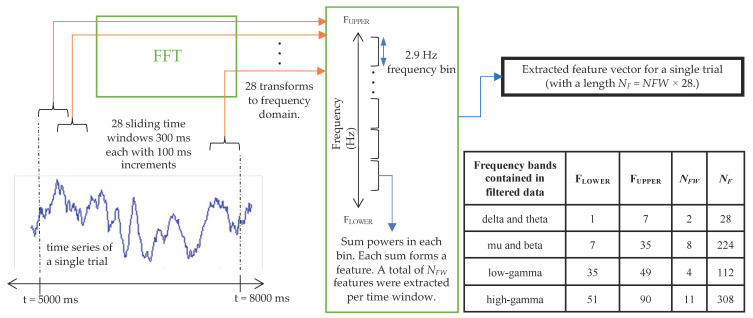
The time–frequency feature extraction algorithm. F_UPPER_ and F_LOWER_, respectively, denote the upper and lower frequency limits. These, in turn, depended on the type of bandpass filter that was applied to the data, as explained in Section 2.3. The implemented values of F_UPPER_ and F_LOWER_ are summarised in the bottom right of the table. The number of features extracted for each frequency range is also summarised in the table.

A time–frequency (TF) algorithm was employed to extract time-based changes in the spectral power of the signals. This technique, explained graphically in Figure 5, was applied to each trial of each IC of each dataset of each participant.

The time range from t = 5000 ms to t = 8000 ms was considered to include signals recorded when wrist movements were sustained at 15% of MVC (during S3, shown in Figure 3). An overlapping sliding window of 300 ms was applied in increments of 100 ms. A fast Fourier transform (FFT) was used to compute the power spectrum for each time window. The resulting power spectrum was then split into equal bands—each 2.9 Hz wide. The sum of the powers within each band formed a feature [59]. Twenty-eight time windows were extracted over the time range considered. The total number of computed features per trial—forming a feature set—is summarised in Figure 5 and depended on the number of frequency bins that fit between the upper and lower frequency limits.

#### 2.4.2. Feature Selection

The dimensionality of the feature sets was reduced using the Bhattacharyya distance (BD). The BD was used to measure how well each single feature differentiated between two classes: between RH and LH motor tasks for the RLI, and between WE and WF motor tasks for the EFI. The BD feature selection method was selected based on success in previous EEG-based BCI studies [46,48,60,61] and its independence from the classifier type [62].

For each feature within a feature set, values from all single trials associated with a specific class (e.g., RH) were utilised to generate a probability density distribution. The same method was carried out for the other class (e.g., LH). A smaller overlap between the two probability density distributions—and greater class separability—resulted in a larger computed BD, according to Equation (1). [63]. Variables *a* and *b* represent the probability distributions of the two classes concerned, while *x* denotes the amplitude of the given feature. In this way, a BD was calculated for each feature in the feature set.

(1) $$BD\left(a,b\right)=-\ln\left(\int \sqrt{a\left(x\right)b\left(x\right)}.dx\;\right)$$

The 20 features with the largest BDs in each dataset were selected to form smaller subsets of features [46,60]. The number of features selected was not extensively optimised, since the purpose of classification in this study was not to maximise the accuracies. Optimisation was conducted exclusively on the mu and beta dataset for participant 1 by comparing the accuracies from feature subsets containing 10, 15, 20, 30, and 40 selected features. The use of 20 selected features was found to produce the best accuracy in this case. In Mohamed et al.’s study (2011) [46], similar feature extraction, BD feature selection, and MD-based classification methods were used for classification between unilateral wrist and finger movements. The authors found that 18 selected TF features (extracted from the mu and beta bands) were optimal. This is similar to the number of TF features selected in this study. Hence, 20 TF features were selected for each trial within each of the 12 datasets from all participants, to simplify the analysis.

#### 2.4.3. Mahalanobis Distance Clustering Classification

The Mahalanobis distance-based classifier is simple and robust and has shown good performance in BCI research [21,46,47,64]. This classifier is based on the Mahalanobis distance (MD), which takes into account the correlation in the data [65]. It is an objective means of classification—that is, it is not susceptible to overtraining, unlike other classifiers, such as artificial neural networks [64]. It can be used to find the dissimilarity between multivariate feature vectors from different classes [65], thus determining if the selected features capture the separateness of the classes. Hence, it was chosen as the only type of classifier in this study, since our focus was not on maximising the accuracies but rather on evaluating the motor control information contained in different frequency bands.

Each single trial was represented as a vector of 20 features. The feature vectors from all trials from each class formed two clusters. Seventy percent of all single trials were used as training data to compute the mean of each cluster/class, while the remaining thirty percent formed the test dataset. The squared MDs (*d_i,cl_*^2^) between each trial in the test set (*p_i_*) and the means of each cluster/class (*m_cl_*) were computed using Equation (2). Subscript *cl* denotes the class type, i.e., either RH, LH, WE, or WF; subscript *i* denotes the trial number; *C_m_*^−1^ is the inverse covariance matrix of vector *m_cl_*; *T* is the transpose operator [66]. A test trial was assigned a predicted class label based on the class linked to the smaller of the two squared MDs. The predicted class label for each trial was then compared with its actual class label to assess the number of correctly classified trials. This comparison was used to calculate the classification accuracy, as shown in Equation (3). This test procedure was repeated, and the resulting accuracies were averaged—using 100-fold cross-validation—for each of the 12 datasets of each participant.

(2) $$d_{i,cl}^{2}={\left(p_{i}-m_{cl}\right)}^{T}C_{m}^{-1}\left(p_{i}-m_{cl}\right)$$

(3) $$Accuracy=\frac{number\;of\;correctly\;classified\;trials}{total\;number\;of\;trials}$$

Prior to splitting the trials into test and training data, outliers were removed; each feature was normalised across all trials, and the order or the trials were randomised.

The MD was also used to remove outlying single trials [65]. To identify outliers, the MD^2^ from each trial’s feature vector (*p_i_*) to the mean of its own cluster/class (*m_cl_*) was calculated according to Equation (2). This formed a set of MDs for all trials [65], from which a standard deviation was computed. If the MD of a trial away from the mean of its cluster was further than three times the value of this standard deviation, then the trial was defined as an outlier and removed [17]. Outliers in the EEG data may have resulted from noisy bursts, recording errors, or signals from unwanted physiological artifacts [64,67].

#### 2.4.4. Determining the Best-Performing IC

For each bandpass-filtered dataset, ICs yielding accuracies greater than a threshold value were selected into a subset of ICs. The threshold values used for the RLI and EFI were 0.7 and 0.59, respectively. These thresholds were chosen to be approximately 10% lower than the lowest accuracies of studies similar to the RLI and EFI (shown in Table 1). For some datasets for the EFI, using these threshold values did not produce any ICs. In these cases, the threshold values were lowered to 0.55, which was 5% higher than the chance level (an accuracy of 0.5).

The spatial activity of each IC from the abovementioned subset of ICs was manually inspected using topographical maps from EEGLAB. The IC with the highest accuracy that also exhibited spatial activity in proximity to the ROIs (SMA, right and left M1-H, right and left PMv, and right and left PFC) was determined to be the best-performing IC.

#### 2.4.5. Analysis of ROIs

As part of our secondary objective, we aimed to validate that motor control information originated from multiple ROIs. Furthermore, we investigated which ROIs were most active in this study.

**Figure 6:**
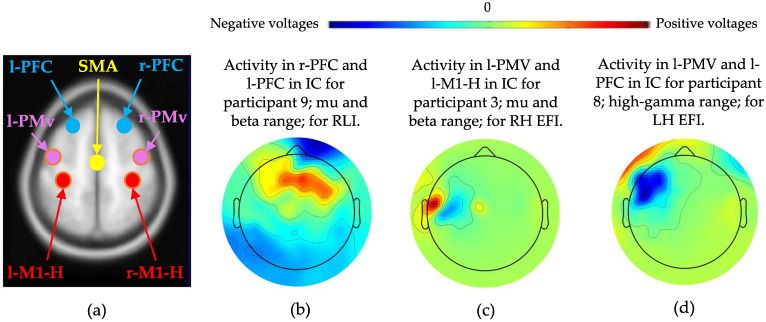
Localisations of neural activity shown in IC topographical maps were compared with typical anatomical positions of the ROIs, shown in (**a**) [42]. The ROI abbreviations are prefixed with “r-” and “l-” to denote right and left. (**b**–**d**) Examples of ICs with accuracies above the thresholds.

Threshold accuracies of 77% and 67.5% were employed to indicate suitable classification rates for the RLI and EFI, respectively. Both values corresponded to the lowest mean accuracies from similar studies in the literature, as listed in Table 1 [18,20]. We identified which of our best-performing ICs produced participant- and frequency-specific accuracies greater than the thresholds. The ROI activity approximated by these ICs was visually identified by comparing EEGLAB’s 2D topographical maps of these ICs to a plot of the typical positions of the ROIs, as shown in Figure 6. We then summed the number of identified ROIs per participant. We also summed the number of observations of each ROI activation across all participants.

#### 2.4.6. Frequency Band Analysis of the Classification Accuracies

For each participant, the accuracies from the best ICs from all four frequency bands were compared. The frequency band producing the maximum of the four accuracies was identified. Each participant was then categorised according to which of the four frequency bands yielded the highest participant-specific accuracy. The number of participants within each of these four categories was computed and plotted for the RLI and EFI. This plot was then used in conjunction with the frequency-specific mean accuracies to assess the discriminatory abilities of features from the four frequency bands.

## 3. Results

Since the LH data for participants 13 and 14 were not usable, the analyses and results for the RLI and LH EFI involved a total of 12 participants. “N/A” in this section indicates where the results are not available. The RH EFI analyses utilised a total of 14 participants.

**Figure 7:**
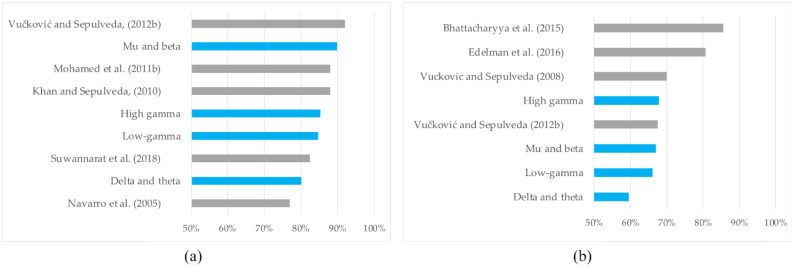
Comparison of the mean accuracies from Table 3 and Table 4 in this study (blue) and mean classification accuracies in the literature shown in Table 1 (grey) for (**a**) the RLI and (**b**) the EFI. “Maximum of all frequency bands” denotes the means of the maximum participant-specific accuracies, considering all frequency bands [13,17,18,19,20,21,22,23].

The accuracies for the best-performing ICs for all datasets and all participants are shown in Table 3 and Table 4. A comparison between the mean accuracies from these tables and the mean accuracies from similar studies (listed in Table 1) is shown in Figure 7. This figure depicts the frequency-specific mean accuracies, as well as the means of the maximum participant-specific accuracies (considering all frequency bands). The highest frequency-specific accuracy for the RLI was 89.88% (SD 6.39%), which was achieved using features extracted from the mu and beta EEG bands, as shown in Table 3. For the EFI, the highest frequency-specific accuracy was 67.85% (SD 5.98%) using features extracted from the high-gamma EEG band, as shown in Table 4. The means of the maximum participant-specific accuracies were 90.98% (SD 5.78%) for the RLI (Table 3) and 69.80% (SD 6.06%) for the EFI (Table 4). The standard deviations for the RLI and EFI ranged between 2.25% and 7.99%, as shown in Table 3 and Table 4.

**Figure 8:**
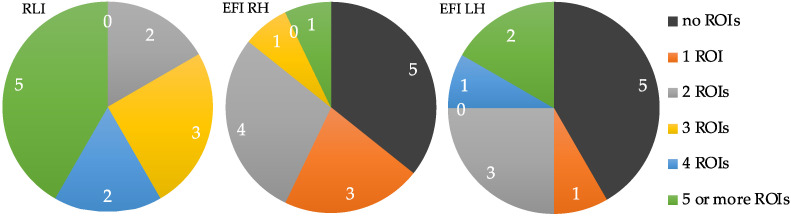
The number of ROIs activated per participant (using the T1 values from Table 5 and Table 6). The values in the charts depict the number of participants per category.

**Figure 9:**
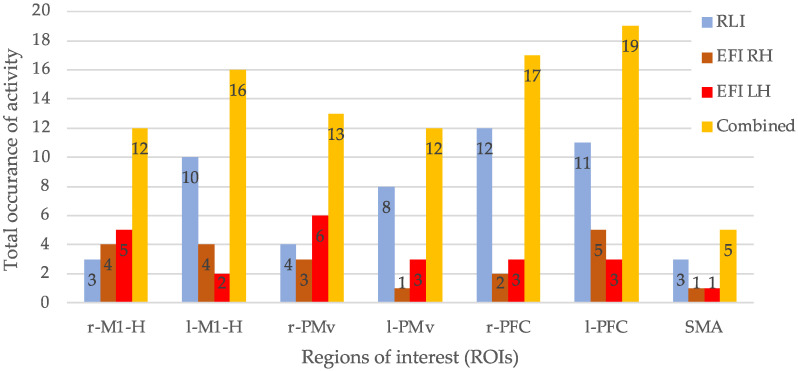
T2 values from Table 5 and Table 6 by ROI for the RLI, EFI, and in combination (sum across the RLI and EFI).

The results of the ROI analysis are shown in Table 5 and Table 6 and Figure 8 and Figure 9. “T1” denotes the total sum of the number of ROIs showing discriminatory activity for each participant. “T2” denotes the total sum of occurrences of discriminatory activity presented in each ROI, considering all participants (abbreviated as “P” in Table 5 and Table 6).

Figure 8 shows the percentage distribution of “T1” values across all participants for the RLI and EFI. This figure compares the number of ROIs—from each participant—approximated to produce accuracies above the threshold accuracies described in Section 2.4.5. Ten out of the twelve participants showed activity emanating from three or more regions in the RLI, and the other two showed activity emanating from two regions. For the EFI, activity in two or more regions was observed in 5/14 participants for RH and in 6/12 participants for LH. For the EFI, some participants (five for RH and five for LH) did not display activity in any ROIs, since their ICs yielded accuracies below the threshold accuracy of 67.5% (described in Section 2.4.5).

Figure 9 shows the “T2” values for the ROIs for the RLI (Table 5) and the EFI (Table 6)—individually and in combination. This graph indicates neural activity in proximity to all seven ROIs, i.e., SMA and bilateral M1-H, PMv, and PFC. The combined (summed) occurrences of activity for both investigations across all participants show that left and right PFC were most frequently activated, with 17 and 19 occurrences, respectively. Right PMv, left PMv, right M1-H, and left M1-H also displayed frequent occurrences, with 13, 12, 12, and 16 combined occurrences, respectively. The SMA was the least activated, showing only five occurrences. Activations in M1-H, PMv, and PFC were both contralateral and bilateral.

**Figure 10:**
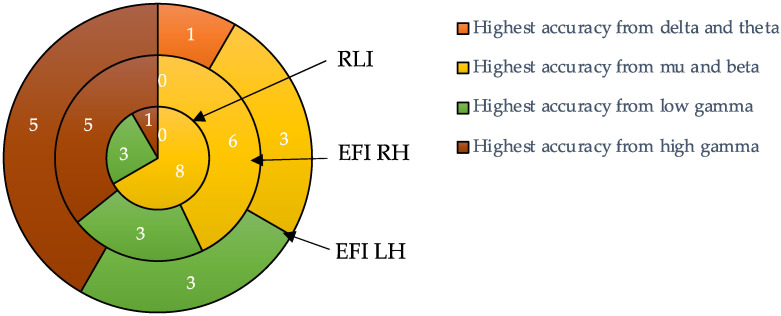
Count and proportion of occurrences of the highest participant-specific accuracies. The colours depict occurrences generated by each of the four frequency bands.

The pie charts in Figure 10 depict the number of occurrences with which each band achieved the highest participant-specific accuracy, as indicated in bold in Table 3 and Table 4. The mu and beta bands yielded the highest accuracies in 8/12 participants for the RLI. The other four participants had their highest accuracies stemming from either the low- or high-gamma bands. For the EFI, the best accuracies were produced by features from either the low- or high-gamma bands for most participants (8/14 for RH and 8/12 for RH). The delta band yielded the lowest count of highest per-participant accuracies, i.e., one participant for LH EFI.

## 4. Discussion

We investigated the discrimination of EEG data associated with real, regulated, isometric wrist extension (WE) and wrist flexion (WF) movements. The investigation included two classification experiments found in previous EEG studies of similar wrist movements (summarised in Table 1). The first experiment involved the discrimination of right-hand (RH) from left-hand (LH) wrist movements—with WE and WF bundled together—which we denoted as the right vs. left investigation (RLI). The second denoted the extension vs. flexion investigation (EFI) and involved a discrimination of unilateral WE and WF movements for both hands. The means of the maximum participant-specific accuracies—90.98% and 69.80% for the RLI and EFI, respectively—are well above the chance level of 50%. These values are also within the range of accuracies obtained in similar BCI investigations involving WE and WF discrimination, as shown in Figure 7. Hence, for the primary objective of this study, we demonstrated successful discrimination between real, regulated, isometric WE and WF motor tasks performed on different limbs (in the RLI) and on the same limb (in the EFI). The lower accuracies of the EFI in comparison to the RLI are expected, since unilateral hand movement discrimination is more challenging than discriminating between movements on separate limbs. The former relies on signals from the same hemispheric cortical regions, while the latter can leverage signals from spatially separable cortical regions [16,17].

For the RLI, we observed frequency-specific mean accuracies above 80% for all four frequency bands, as shown in Table 3. These accuracies were all above the chance level and comparable to accuracies from similar studies, as shown in Figure 7a. This suggests that the delta, theta, mu, beta, low-gamma, and high-gamma frequencies all contained sufficient motor control information to enable the discrimination of the WE and WF motor tasks performed on different limbs. Similarly, the EFI mean frequency-specific accuracies were above 66%—as shown in Table 4—for the mu and beta, low-gamma, and high-gamma bands, and were similar to the mean accuracies in two former studies [17,20] shown in Figure 7b. This suggests that these frequencies hold identifiable movement-related neural control information for the unilateral discrimination of WE and WF motor tasks.

For both the RLI and EFI, signals representing neural activity from all seven ROIs produced features that yielded classification accuracies comparable to mean accuracies from two similar BCI investigations [18,20]. This suggests that the seven chosen ROIs contain sufficient motor control information that can differentiate between bilateral and unilateral WE and WF motor tasks. Furthermore, this motor control-related neural information originated from multiple ROIs for a significant number of participants.

The abovementioned performances of the frequency- and ROI-related discrimination experiments addressed our secondary objective. These results indicated that neuromotor control patterns differentiating the kinematic differences between real, regulated, isometric WE and WF motor tasks include information from multiple EEG frequency bands and multiple ROIs. This suggests that BCI studies involving WE and WF (as well as other hand movements) should explore signals and features emanating from all EEG frequency bands and from multiple ROIs, including SMA and bilateral M1-H, PMv, and PFC. This challenges common methods many sensorimotor BCI studies employ that limit signal analysis and feature extraction to contralateral M1-H and mu and beta rhythms [14,15].

We found that features from the mu and beta bands played the most prominent role in discriminating between right and left wrist movements. This was deduced from two results associated with these bands within the RLI: (1) the highest frequency-specific mean accuracy (67.85% shown in Table 3) and (2) the largest proportion of maximum participant-specific accuracies (8/12 shown in Figure 10). Vučković and Sepulveda (2012) [20] also found that features from mu and beta were dominant in the classification between right and left imagined WE and WF. BCI studies have commonly relied on mu and beta sensorimotor rhythms for feature extraction when classifying right and left hand movements [14].

For the unilateral discrimination of WE and WF movements, some studies have demonstrated the importance of the mu and beta bands [22,23], while others have favoured the delta and gamma bands [17,20]. Our study corroborates the importance of the mu, beta, and gamma bands for this type of BCI experiment but contrarily found the delta and theta bands to be least valuable. This may be due to differences between our methods and those used in the abovementioned studies, but it may also be due to differences in the types of WE and WF motor tasks. Our regulated (MVC-normalised, isometric) motor tasks may have been comparatively more complex to execute [24], requiring more concentration and thus recruiting more gamma activity [68]. This theory is supported by our best EFI results stemming from the gamma bands (high gamma in Table 4 and a combination of low and high gamma in Figure 10).

Complex motor tasks that require a high level of attention and continuous online monitoring and coordination have been widely associated with neural activity in the PFC [27,69,70,71]. The prevalence of activity in bilateral PFC in our study—shown in Figure 9—could have been responsible for monitoring and coordinating the correctness of the regulated, isometric wrist movements. We also observed pronounced activity from bilateral M1-H and PMv. These regions, as well as SMA, have been linked to hand movement control in several neuroscience studies [32,35,69,70]. PMv and SMA have been linked to sensorimotor integration and movement execution and planning [70]. Furthermore, signals approximating activity from M1-H are commonly used for feature extraction in BCI studies [14]. To elucidate the interactions/relationships between these seven cortical regions in controlling WE and WF, further research is required.

Dynamic causal modelling has been employed to explore the causal, cross-frequency communication (spanning multiple EEG frequency bands) between multiple ROIs in controlling hand motor tasks [32,34,35,43,69,70]. Future work will use this technique to elucidate the control of real, regulated, isometric WE and WF.

In addition, we will seek to improve the classification of these motor tasks by exploring other feature extraction, selection, and classification methods. We plan to explore the combination of time–frequency features from multiple frequency bands, as well as the use of phase-based features, statistical features, and connectivity measures [11]. The advantages and disadvantages of other feature selection methods will also be explored. These methods include the Davies–Bouldin index [20], Kullback–Leiber divergence, and analysis of variance (ANOVA) [72]. Furthermore, we plan to compare the classification performances of MD clustering with other classifiers, including artificial neural networks, support vector machines, linear discriminant analysis, and deep learning [48,73,74]. The use of the abovementioned feature selection and classification methods could also be investigated using wrapper approaches [62,74].

EEG or BCI studies exploring hand motor control typically involve between 5 and 20 participants [13,20,23,70,75,76]. The high mental demand on participants during data recording and the large inter-participant variance generally limit the number of participants recruited in each study [10,64]. In this study, we were additionally constrained to 14 participants due to the cost and time associated with recording 128-channel data using the Brain Products system. This may limit the generalisability of the results. BCI studies typically address this common issue by conducting analyses on a per-participant basis [10,64]. Our study followed suit and showed consistent results across participants, with standard deviations ranging between 2.25% and 7.99%. In comparison, the standard deviation ranges from two similar studies were 5–14% and 9.7–11.26% [13,20]. Our lower inter-participant variance in accuracies suggests that our methods and results may be generalisable. This could be validated by repeating our study on a new cohort of 15–20 participants. Additionally, we plan on exploring transfer learning as a popular emerging approach for dealing with inter-participant variance and limited training data [74].

## 5. Conclusions

An effective EEG-based BCI controlling a bionic hand requires reliable and accurate interpretation of signals associated with the neural control of key hand movements. In pursuit of this goal, this study demonstrated sufficiently accurate discrimination of real, regulated, isometric WE and WF movements—bilaterally and unilaterally. Our study also demonstrated that all EEG frequencies and seven cortical regions contained sufficient motor control information to enable this discrimination. The seven cortical regions consisted of the hand homunculus of the primary cortex, the ventral premotor cortex, the supplementary motor area, and the prefrontal cortex. The analysis of full-frequency signals from these seven cortical regions could lead to improved EEG interpretation in future sensorimotor BCI studies.

## Figures and Tables

**Table 1:** Review of EEG-based BCI studies of WE and WF motor tasks. The green rows include studies similar to the RLI, while the orange rows include studies similar to the EFI. WS and WP refer to wrist supination and wrist pronation, respectively.

Type of Wrist Motor Tasks Classified	Classification Accuracy	Prominent Frequencies	Reference
RH vs. LH groupings of WE, WF, WS, and WP; real and imagined movements	77% mean across participants using independent component analysis	Theta, mu, and beta	[18]
RH vs. LH WE; imagined movements RH vs. LH WF; imagined movements	88% mean across participants	Low gamma (<50 Hz)	[19]
RH vs. LH groupings of WE and WF; imagined movements	83% mean across participants	Mu and beta	[13]
RH vs. LH groupings of WE and WF; imagined movements	92% mean across participants	Mu and beta	[20]
RH vs. LH groupings of WE, WF, FF, FE, and TR; real and imagined movements	88% mean across participants	Mu and beta	[21]
Unilateral WE vs. WF; real and imagined movements	70% and 80% means across participants (real and imagined, respectively)	Delta	[17]
Unilateral WE vs. WF; imagined movements	67.5% mean across participants	Delta, low gamma, and high gamma (>60 Hz)	[20]
Unilateral WE vs. WF; imagined movements	85.48% mean across participants	Mu and beta	[22]
Unilateral WE vs. WF vs. WS vs. WS; RH imagined movements	80.6% mean considering only WE and WF classes	Mu and beta	[23]

**Table 3:** Percentage accuracy for the best-performing IC in the RLI. Bold numbers indicate the highest participant-specific accuracies.

Participant Number	Delta and Theta (1–7 Hz)	Mu and Beta (8–35 Hz)	Low Gamma (36–49 Hz)	High Gamma (51–90 Hz)	Maximum of All Bands
1	74.53	**90.00**	89.22	90.00	90.00
2	65.84	**81.36**	80.34	70.54	81.36
3	80.60	**91.88**	88.02	89.32	91.88
4	81.60	85.32	**87.56**	85.13	87.56
5	83.35	91.04	**92.41**	88.26	92.41
6	79.47	**94.44**	83.88	84.06	94.44
7	78.93	**99.74**	92.30	94.16	99.74
8	82.04	**87.79**	77.48	80.18	87.79
9	87.91	**99.44**	87.76	91.75	99.44
10	81.09	**87.25**	83.57	78.17	87.25
11	78.57	78.66	**82.69**	78.38	82.69
12	87.68	91.65	71.83	**93.64**	93.64
Mean (SD)	80.13 (5.84)	**89.88** (6.39)	84.76 (6.10)	85.30 (7.27)	90.68 (5.78)

**Table 4:** Percentage accuracy for the best-performing IC for the EFI of RH and LH movements. Bold numbers indicate the highest participant-specific accuracies. “N/A” = not available.

Participant Number	Delta and Theta (1–7 Hz)		Mu and Beta (8–35 Hz)		Low Gamma (36–49 Hz)		High Gamma (51–90 Hz)		Maximum of All Bands	
	RH	LH	RH	LH	RH	LH	RH	LH	RH	LH
1	55.50	63.45	62.03	65.08	62.07	**65.52**	**66.67**	63.21	66.67	65.52
2	55.88	56.71	66.55	61.38	66.03	61.38	**68.93**	**66.61**	68.93	66.61
3	59.38	58.06	**78.35**	65.33	72.91	**67.97**	78.14	65.83	78.35	67.97
4	59.62	74.50	**76.12**	**79.80**	74.91	75.67	74.85	78.41	76.12	79.80
5	57.58	56.59	58.02	57.64	**63.00**	58.79	60.15	**62.82**	63.00	62.82
6	55.58	56.25	59.46	57.67	**63.67**	62.02	62.48	**62.91**	63.67	62.91
7	62.61	67.03	79.27	**69.44**	**80.15**	67.48	76.38	68.93	80.15	69.44
8	56.93	56.20	**71.00**	**74.05**	67.27	70.16	69.47	71.79	71.00	74.05
9	57.04	56.21	58.00	61.76	59.56	62.48	**60.40**	**71.05**	60.40	71.05
10	56.31	57.96	**74.29**	62.31	62.50	**64.12**	67.25	59.05	74.29	64.12
11	56.90	**75.41**	55.38	68.83	58.52	63.65	**60.19**	66.86	60.19	75.41
12	55.90	57.20	**68.31**	73.11	64.59	72.72	65.10	**76.00**	68.31	76.00
13	61.64	N/A	**71.80**	N/A	66.67	N/A	64.14	N/A	71.80	N/A
14	58.26	N/A	67.39	N/A	65.64	N/A	**76.59**	N/A	76.59	N/A
Mean (SD)	57.79 (2.25)	61.30 (7.20)	67.57 (7.99)	66.37 (6.86)	66.25 (6.01)	66.00 (4.98)	**67.91** (6.40)	**67.79** (5.72)	69.96 (6.61)	69.64 (5.64)
Mean (SD)	59.55 (5.35)		66.79 (7.37)		66.12 (6.01)		**67.85** (5.98)		69.80 (6.06)	

**Table 5:** The presence of neural activity in cortical ROIs approximated from the 2D plots of ICs that produced features yielding accuracies greater than 77% (for the RLI). An “x” indicates the presence of activity. The prefixes “r-” and “1-” in the ROI abbreviations of the header row denote the right and left cortical regions, respectively. “P” in the first column is used as an abbreviation for participant.

P	r-M1-H	l-M1-H	r-PMv	l-PMv	r-PFC	l-PFC	SMA	T1
1					x	x		2
2		x			x			2
3		x			x	x		3
4		x			x	x		3
5		x	x	x	x	x		5
6		x		x	x	x		4
7				x	x	x		3
8	x	x	x	x	x	x		6
9	x	x	x	x	x	x	x	7
10		x		x	x	x		4
11		x		x	x	x	x	5
12	x	x	x	x	x	x	x	7
T2	3	10	4	8	12	11	3	51

**Table 6:** The presence of neural activity in cortical ROIs approximated from the 2D plots of ICs that produced features yielding accuracies greater than 67.5% (for the EFI). An “x” indicates the presence of activity. The prefixes “r-” and “1-” in the ROI abbreviations of the header row denote the right and left cortical regions, respectively. “P” in the first column is used as an abbreviation for participant. “N/A” = not available.

P	r-M1-H		l-M1-H		r-PMv		l-PMv		r-PFC		l-PFC		SMA		T1	
Hand	RH	LH	RH	LH	RH	LH	RH	LH	RH	LH	RH	LH	RH	LH	RH	LH
1															0	0
2			x								x				2	0
3			x		x		x		x		x		x	x	6	1
4	x	x		x	x	x		x		x	x	x			3	6
5															0	0
6															0	0
7	x	x				x									1	2
8	x				x	x		x		x		x			2	4
9		x				x									0	2
10			x												1	0
11		x		x		x		x		x		x			0	6
12	x	x				x									1	2
13		N/A		N/A		N/A		N/A	x	N/A	x	N/A		N/A	2	0
14		N/A	x	N/A		N/A		N/A		N/A	x	N/A		N/A	2	0
T2	4	5	4	2	3	6	1	3	2	3	5	3	1	1	20	23

## Data Availability

Force and EEG data are unavailable due to privacy or ethical restrictions.
